# Supplementary material for: Mutations in PCYT2 disrupt etherlipid biosynthesis and cause a complex hereditary spastic paraplegia
Source: Brain. 2019 Oct 22;142(11):3382–97. doi: 10.1093/brain/awz291 (PMC6821184; doi:10.1093/brain/awz291)
Supplement: awz291_Supplementary_Data [file awz291_supplementary_data.zip › awz291-Suppl_data/Supplementary_Data4.zip!/Supplementary information 4 - plasma lipidomics.zip!/Plasma lipidomics/Control vs Patient/Statistical plots and data/TG species/Heatmap.pdf]

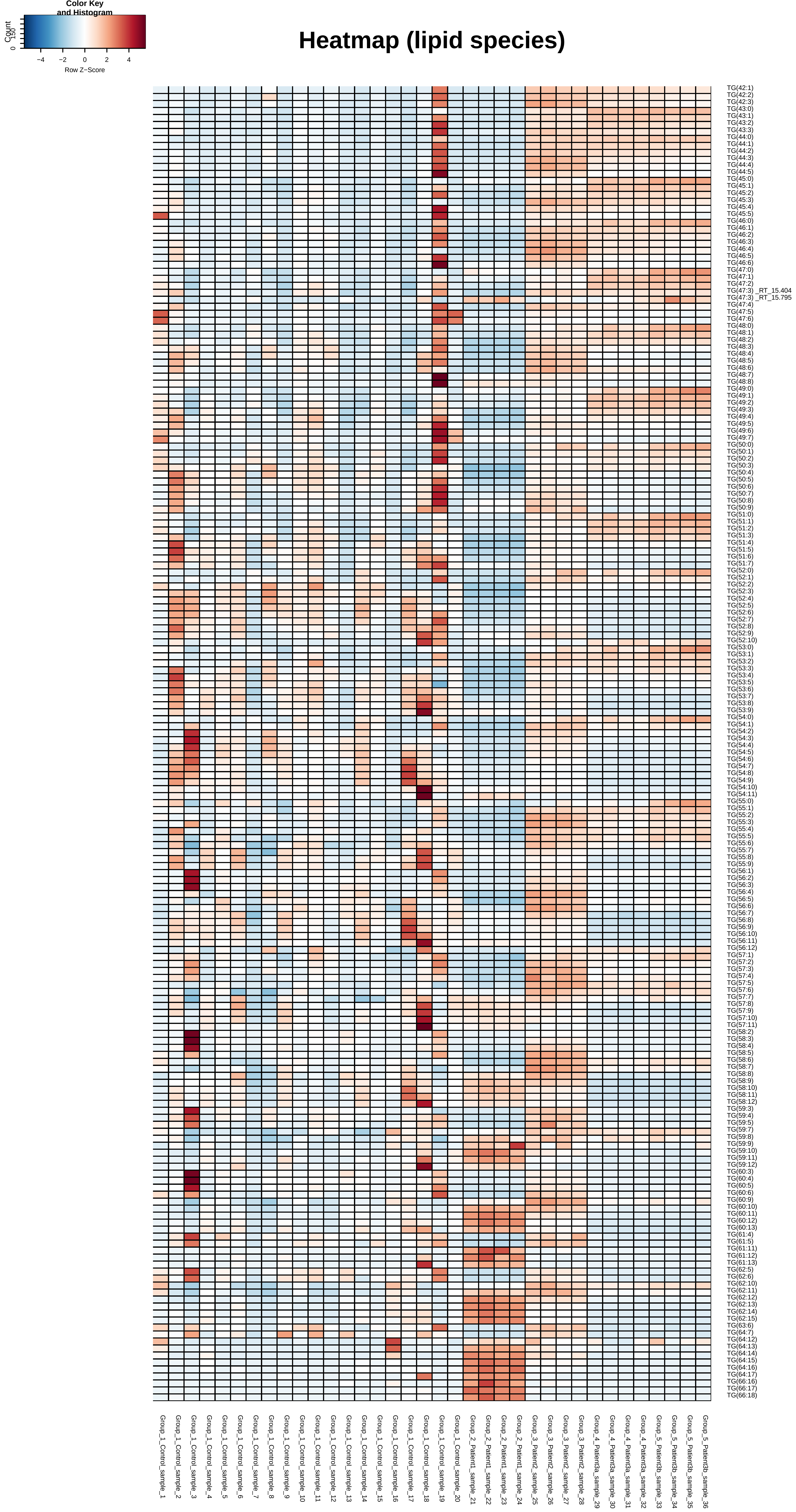
Color Key
and Histogram
Count
0
150
−4
−2
0
2
4
Row Z−Score
Heatmap (lipid species)
TG(42:1)
TG(42:2)
TG(42:3)
TG(43:0)
TG(43:1)
TG(43:2)
TG(43:3)
TG(44:0)
TG(44:1)
TG(44:2)
TG(44:3)
TG(44:4)
TG(44:5)
TG(45:0)
TG(45:1)
TG(45:2)
TG(45:3)
TG(45:4)
TG(45:5)
TG(46:0)
TG(46:1)
TG(46:2)
TG(46:3)
TG(46:4)
TG(46:5)
TG(46:6)
TG(47:0)
TG(47:1)
TG(47:2)
TG(47:3) _RT_15.404
TG(47:3) _RT_15.795
TG(47:4)
TG(47:5)
TG(47:6)
TG(48:0)
TG(48:1)
TG(48:2)
TG(48:3)
TG(48:4)
TG(48:5)
TG(48:6)
TG(48:7)
TG(48:8)
TG(49:0)
TG(49:1)
TG(49:2)
TG(49:3)
TG(49:4)
TG(49:5)
TG(49:6)
TG(49:7)
TG(50:0)
TG(50:1)
TG(50:2)
TG(50:3)
TG(50:4)
TG(50:5)
TG(50:6)
TG(50:7)
TG(50:8)
TG(50:9)
TG(51:0)
TG(51:1)
TG(51:2)
TG(51:3)
TG(51:4)
TG(51:5)
TG(51:6)
TG(51:7)
TG(52:0)
TG(52:1)
TG(52:2)
TG(52:3)
TG(52:4)
TG(52:5)
TG(52:6)
TG(52:7)
TG(52:8)
TG(52:9)
TG(52:10)
TG(53:0)
TG(53:1)
TG(53:2)
TG(53:3)
TG(53:4)
TG(53:5)
TG(53:6)
TG(53:7)
TG(53:8)
TG(53:9)
TG(54:0)
TG(54:1)
TG(54:2)
TG(54:3)
TG(54:4)
TG(54:5)
TG(54:6)
TG(54:7)
TG(54:8)
TG(54:9)
TG(54:10)
TG(54:11)
TG(55:0)
TG(55:1)
TG(55:2)
TG(55:3)
TG(55:4)
TG(55:5)
TG(55:6)
TG(55:7)
TG(55:8)
TG(55:9)
TG(56:1)
TG(56:2)
TG(56:3)
TG(56:4)
TG(56:5)
TG(56:6)
TG(56:7)
TG(56:8)
TG(56:9)
TG(56:10)
TG(56:11)
TG(56:12)
TG(57:1)
TG(57:2)
TG(57:3)
TG(57:4)
TG(57:5)
TG(57:6)
TG(57:7)
TG(57:8)
TG(57:9)
TG(57:10)
TG(57:11)
TG(58:2)
TG(58:3)
TG(58:4)
TG(58:5)
TG(58:6)
TG(58:7)
TG(58:8)
TG(58:9)
TG(58:10)
TG(58:11)
TG(58:12)
TG(59:3)
TG(59:4)
TG(59:5)
TG(59:7)
TG(59:8)
TG(59:9)
TG(59:10)
TG(59:11)
TG(59:12)
TG(60:3)
TG(60:4)
TG(60:5)
TG(60:6)
TG(60:9)
TG(60:10)
TG(60:11)
TG(60:12)
TG(60:13)
TG(61:4)
TG(61:5)
TG(61:11)
TG(61:12)
TG(61:13)
TG(62:5)
TG(62:6)
TG(62:10)
TG(62:11)
TG(62:12)
TG(62:13)
TG(62:14)
TG(62:15)
TG(63:6)
TG(64:7)
TG(64:12)
TG(64:13)
TG(64:14)
TG(64:15)
TG(64:16)
TG(64:17)
TG(66:16)
TG(66:17)
TG(66:18)
Group_1_Control_sample_1
Group_1_Control_sample_2
Group_1_Control_sample_3
Group_1_Control_sample_4
Group_1_Control_sample_5
Group_1_Control_sample_6
Group_1_Control_sample_7
Group_1_Control_sample_8
Group_1_Control_sample_9
Group_1_Control_sample_10
Group_1_Control_sample_11
Group_1_Control_sample_12
Group_1_Control_sample_13
Group_1_Control_sample_14
Group_1_Control_sample_15
Group_1_Control_sample_16
Group_1_Control_sample_17
Group_1_Control_sample_18
Group_1_Control_sample_19
Group_1_Control_sample_20
Group_2_Patient1_sample_21
Group_2_Patient1_sample_22
Group_2_Patient1_sample_23
Group_2_Patient1_sample_24
Group_3_Patient2_sample_25
Group_3_Patient2_sample_26
Group_3_Patient2_sample_27
Group_3_Patient2_sample_28
Group_4_Patient3a_sample_29
Group_4_Patient3a_sample_30
Group_4_Patient3a_sample_31
Group_4_Patient3a_sample_32
Group_5_Patient3b_sample_33
Group_5_Patient3b_sample_34
Group_5_Patient3b_sample_35
Group_5_Patient3b_sample_36
